# The Role of Oral Probiotics in Alleviating Inflammation, Symptom Relief, and Postoperative Recurrence and Their Side Effects in Adults With Crohn's Disease: A Systematic Review

**DOI:** 10.7759/cureus.50901

**Published:** 2023-12-21

**Authors:** Devendar Banoth, Muhammad Hassaan Wali, Khava Bekova, Noor Abdulla, Simhachalam Gurugubelli, Yi Mon Lin, Safeera Khan

**Affiliations:** 1 Internal Medicine, California Institute of Behavioral Neurosciences and Psychology, Fairfield, USA

**Keywords:** lactobacillus species, inflammatory bowel disease, crohn's disease, probiotics, crohn's disease remission

## Abstract

Crohn's disease (CD) is a lifelong problem for patients, despite having multiple pharmacological options and surgeries for treatment. In order to achieve best results, probiotics are being used even though their efficacy is still debatable. This systematic review analyzes the safety and efficacy of several probiotics in CD. PubMed, the Cochrane Library, and ScienceDirect are the databases searched for randomized controlled trials (RCTs), animal studies, in vitro studies, and reviews. After quality appraisal and cross checking the literature, this systematic review is carried out grounded on Preferred Reporting Items for Systematic Review and Meta-Analysis 2020 (PRISMA 2020) guidelines. A study of 16 papers in total which include nearly 2023 subjects showed that only very few probiotics are efficient in furnishing remission in CD complaints. Kefir, an inexpensive fermented milk product, significantly reduced the inflammation and drastically bettered the quality of life and hence can be considered as an asset for CD patients. *Lactobacillus thermophilus*, *Bifidobacterium longum*, *Enterococcus faecalis*, and *Bacillus licheniformis* can control diarrhea in patients of 22-54-year age group and improve cognitive reactivity in sad mood with short-term consumption. VSL#3 (VSL Pharmaceuticals, Gaithersburg, Maryland, United States) has good efficacy in precluding recurrence and easing side effects after ileocecal resection in adults. Animal models and lab studies have proved that *Lactobacillus plantarum CBT LP3*, *Saccharomyces cerevisiae CNCM I-3856* (*yeast*), few strains of *Lactobacillus plantarum*,* Bifidobacterium animalis *spp.,* Lactobacillus acidophilus LA1*,* Lactobacillus paracasei 101/37*, and especially *Bifidobacterium breve Bbr8* are significant enough to ameliorate the disease condition. In conclusion, probiotics are safe in CD with very few modifiable side effects. Some probiotics are proven to be significant in animal and lab studies; hence, these should be studied in human RCTs, to check their efficiency in human beings. There are limited observational and interventional studies in this regard. Large population-sizes trials are highly demanded in the areas of prognosticated positive results that are mentioned in this systematic review.

## Introduction and background

Inflammatory bowel disease (IBD) that includes Crohn's disease (CD) and ulcerative colitis (UC) is a complaint of lifetime and deteriorates the quality of life of patients and their families substantially [1]. 1.3% of US adults, which is three million, were reported to be diagnosed with IBD in 2015 [2]. The prevalence in the United States is estimated at 58 per 100,000 children and 119-241 per 100,000 grown-ups, and it is increasing in both age groups. Although new cases occur in after periods, most cases are diagnosed in the twenties and forties. Prevalence is more in the white race. 6.3 billion dollars is the estimated annual economic burden to the US healthcare system [3-6]. In China from 1994 to 2004, the incidence had increased by threefold [7]. According to the World Journal of Gastroenterology, areas with the highest incidence are the United Kingdom, North America, and the northern part of Europe [8,9]. The prevalence of CD in Europe varies from <10 to about 150 per 100000 occupants [10,11].

In 2017, there were 6.8 million cases of IBD globally, and the age-formalized death rate is 0.51 (0.42-0.54) per 100,000 population. Loftiest age-standardized prevalence rate was seen in high socio-demographic index (SDI) locations. From 1990 to 2017, the prevalence of IBD increased substantially in many regions which might pose a significant social and financial burden on governments and health systems in the coming times [12].

CD, a chronic IBD, can affect any portion of the gastrointestinal tract. It manifests as spastic granulomatous patches due to effects of environmental factors, inheritable predisposition, and mucosal immunity. Common presenting symptoms are abdominal pain, rectal bleeding, weight loss, fever, fatigue, and diarrhea. Uncontrolled inflammation may lead to long-term complications such as fibrotic strictures, enteric fistulae, and intestinal neoplasia. Therefore, early and effective control of inflammation is of paramount value [13,14].

The etiology of CD is multifactorial; thus, understanding only one aspect of IBD pathogenesis does not reflect the complex nature of IBD, nor will it enhance its clinical management. Therefore, it is pivotal to segregate the interactions between the various factors in IBD pathogenesis [15]. Several studies have reported microbial dysbiosis and immune and metabolic dysregulation in IBD cases; however, this data is not sufficient to produce signatures that can differentiate between complaint relapse and remission. The current knowledge gap in IBD hinders the advancement of clinical decision for treatment, as well as the prediction of disease relapse. There has been exploration going on regarding the use of prebiotics, probiotics, and synbiotics independently and in combination with other drugs like 6-mercaptopurine (6MP), aminosalicylates, corticosteroids, antibiotics, and surgical procedures to ameliorate the condition [15].

The management of CD and UC has changed drastically over the last two decades due to the introduction of targeted biological therapies, but the impact of these new medicines in altering the natural history of disease is still under debate. Latest evidence seems to suggest that their efficacy might be incompletely dependent on the timing of their introduction and further management of the disease. So far, the potential role for a more dynamic approach with treatments grounded on sequencing and combining targeted preventive and curative therapies has been explored only to a minimal extent.

Despite the available treatment strategies, a significant number of cases suffer one or multiple surgeries in their continuance. The current pharmacological treatment has variable efficacy and is associated with the threat of significant side effect; hence, there is a constant need to search for new types of therapies with optimum safety standard [16]. The arrival of biologics has clearly represented a good advance in IBD treatment, but their efficacy in changing the natural history of the disease has not been clearly established [8]. In view of the above factors, we aim to explore in this systematic review the efficacy of probiotics in remission, inflammation, symptom relief, and postoperative recurrence and their side effects in adults with CD based on the recent research. More importantly, we are fastening on the stylish probiotic preparations available with considerable evidence from the latest works, to be recommended for the adult patients in agony suffering from CD.

## Review

Methods

This manuscript is written according to the Preferred Reporting Items for Systematic Review and Meta-Analysis 2020 (PRISMA 2020) guidelines [17]. Table 1 shows the total number of articles available on different databases regarding the role of probiotics in CD.

**Table 1:** Data sources and search strategy

Database	Keywords and search strategy	Number of available papers
PubMed	Probiotics and Crohn's disease	689
PubMed MeSH	"Probiotics"[MeSH] AND ("Probiotics"[MeSH]) AND "Crohn Disease/diet therapy"[MeSH] OR "Crohn Disease/parasitology"[MeSH] OR "Crohn Disease/prevention and control"[MeSH]	457
Cochrane Library	Probiotics and Crohn's disease	92
ScienceDirect	Probiotics and Crohn's disease	5979
Total	Without filters, including duplicates	7217

**Figure 1:**
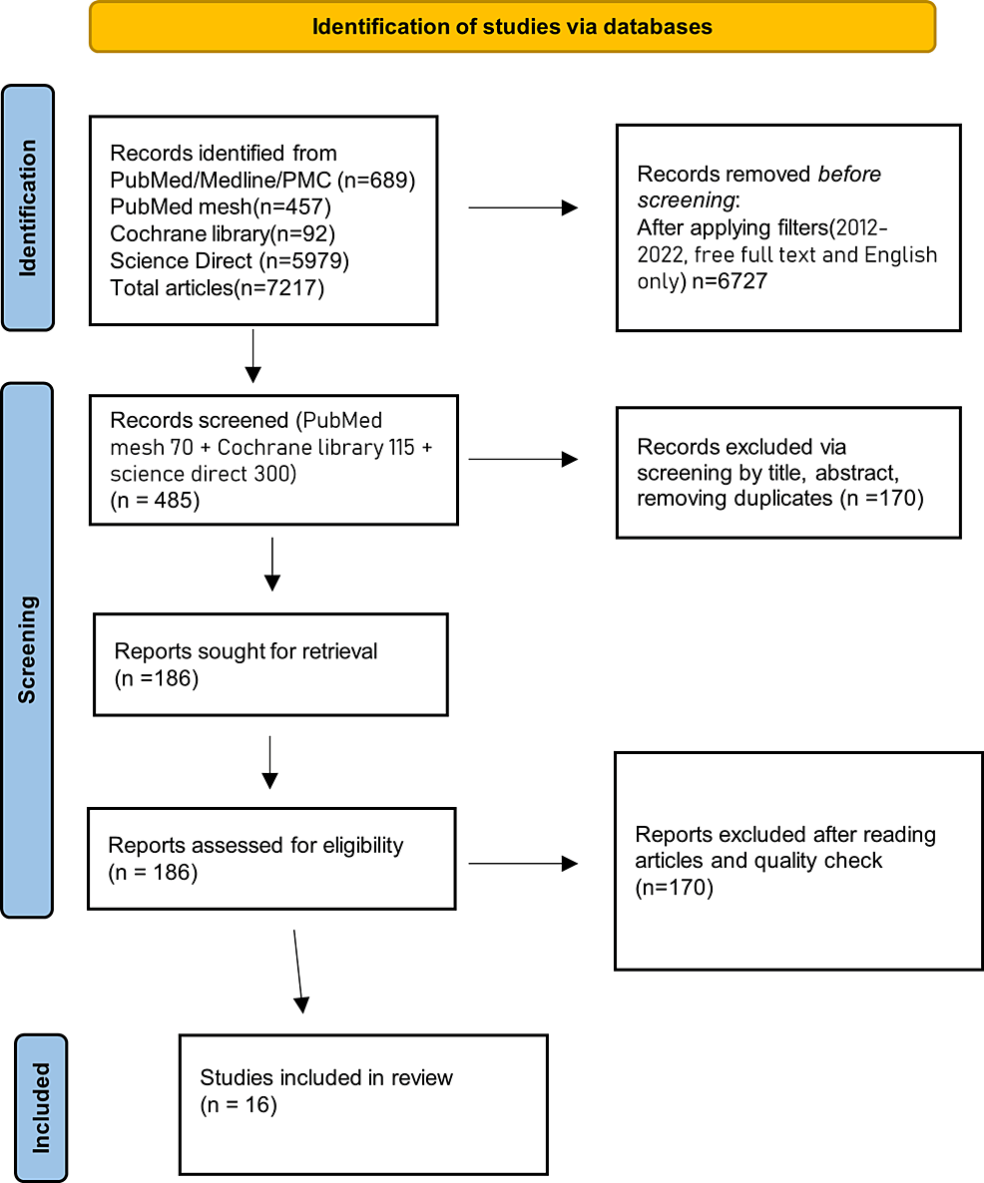
PRISMA study flowchart PRISMA: Preferred Reporting Items for Systematic Review and Meta-Analysis

Figure 1 depicts the study screening and selection.

Inclusion and Exclusion Criteria

Studies comprising pediatric age group patients, pregnant women, and critical cases are removed. Studies on IBD with less focus on CD are removed as well. Probiotics given by other means like fecal transplantation and cancer-related papers are excluded also.

Result

Quality Appraisal

The Cochrane bias assessment tool for randomized controlled trials (RCTs), A MeaSurement Tool to Assess systematic Reviews (AMSTAR) checklist for systematic reviews, the SYstematic Review Centre for Laboratory animal Experimentation (SYRCLE) for animal studies, and the Scale for the Assessment of Narrative Review Articles (SANRA) checklist for other studies are the tools used for quality check. The list of selected studies with the year of publication, study type, and number of subjects involved is presented in Table 2. 

**Table 2:** List of studies and their characteristics RCT: randomized controlled trial; CD: Crohn's disease

Author of study	Year of publication	Type of study	Number of patients/subjects involved
Bjarnason et al. [18]	2019	RCT (double-blind placebo-controlled trial)	250 in batch studies over four years. Each batch studied for four weeks
Yılmaz et al. [19]	2019	RCT (open-label randomized controlled, single-center, prospective trial)	45 subject studies over four weeks
Wu et al. [20]	2021	RCT (single-blind crossover study)	46
Ahmed et al. [21]	2013	RCT (a prospective double-blind randomized crossover study)	8
Fedorak et al. [22]	2014	RCT	119 (59 for intervention, 60 for placebo)
Bourreille et al. [23]	2013	RCT	125 finalized patients (59 for *Saccharomyces boulardii* and 66 for placebo)
Kim et al. [24]	2020	Animal study	18 mice
Sivignon et al. [25]	2015	Animal model and in vitro study	
Choi et al. [26]	2019	Animal model and in vitro study	
Leccese et al. [27]	2020	In vitro study	Blood samples of 18 patients
Chen et al. [28]	2021	Systematic review and meta-analysis	289
Dore et al. [29]	2019	Systematic review and meta-analysis of RCTs	178
Lorentz and Müller [30]	2022	Systematic review	32 subjects after removing duplicates (482 in total, out of which 450 are covered in other studies in this systematic review)
Limketkai et al. [31]	2020	Systematic review	45
Shen et al. [32]	2013	Meta-analysis	43 subjects after removing duplicates (total 349, out of which 306 are covered in other papers of this systematic review)
Zhuang et al. [33]	2020	Systematic review	807 (709 CD patients and 98 controls)
Total			2023 subjects were studied (1987 human subjects, more than 18 mice, and blood samples of 18 patients)

The effects of various probiotics on CD are assessed in this systematic review which include disease remission and symptom relief, improving the quality of life of the patients, effect on Crohn's Disease Activity Index (CDAI), effects on inflammation, emotional symptoms, bowel functions, systemic functions, wellbeing, abdominal pain, abdominal mass, quantity of liquid stools, hemoglobin levels, hematocrit, white blood cell (WBC) counts, erythrocyte sedimentation rate (ESR), C-reactive protein (CRP), calprotectin levels, impact on diarrhea thus on mood disorders, efficacy in altering microflora in colitis, postoperative recurrence, disease relapse in steroid or aminosalicylate users, histopathology, immunomodulation, and side effects. The review of various RCTs, lab and animal studies, and review studies are analyzed in Table 3, Table 4, and Table 5, respectively, that include the sub-topics discussed, types of probiotics used, other drugs given for the studies, and their results along with their side effects.

**Table 3:** RCTs RCTs: randomized controlled trials; CD: Crohn's disease; 5-ASA: 5-aminosalysilic acid; WBC: white blood cell; ESR: erythrocyte sedimentation rate; CRP: C-reactive protein; CDAI: Crohn's Disease Activity Index; AEs: adverse events

Author of study	Year of publication	Sub-topic	Probiotics/intervention studied	Placebo and/or other drugs given	Result and side effects
Bjarnason et al. [18]	2019	Symptom relief and inflammation (emotional symptoms, bowel functions, systemic functions, wellbeing, abdominal pain, abdominal mass, number of liquid stools, hemoglobin, hematocrit, WBC count, ESR, CRP, calprotectin)	Symprove (Farnham, Surrey, United Kingdom) (a mixture of *Lactobacillus rhamnosus NCIMB 30174, Lactobacillus plantarum NCIMB 30173, Lactobacillus acidophilus NCIMB 30175, *and* Enterococcus faecium NCIMB 30176*)	Placebo for randomized controls. Azathioprine and 5-ASA had been taken by some patients in both cases and controls	Except for the WBC count levels, there was no improvement seen in any other area, and the intensity and the speed of raise in calprotectin levels are less in cases than controls; however, no side effects were reported.
Yılmaz et al. [19]	2019	Alterations in fecal microbiota (stool *Lactobacillus*). Symptom relief (abdominal pain, bloating, stool frequency, stool consistency, and feeling good)	Kefir (fermented dairy product that contains *Lactobacillus kefiri, Lactobacillus casei, Lactobacillus kefiranofaciens, Pediococcus acidilactici,*and* Lactococcus lactis*)	None	Fecal *Lactobacillus* levels improved in patients with and without* Lactobacillus* prior to the study significantly (p=0.005), ESR levels decreased, CRP levels dropped (p=0.015), hemoglobin levels improved, bloating decreased in the last two weeks appreciably (p=0.012), abdominal pain had reduced to a great extent, and patients gave appreciable feeling good score (p=0.032). No side effects including symptoms of lactose intolerance were reported.
Wu et al. [20]	2021	Impact on diarrhea, thus on mood disorders	*Lactobacillus thermophilus, Bifidobacterium longum, Enterococcus faecalis, *and *Bacillus licheniformis*	Chamomile capsule 1400 mg that contains terpenoids, flavonoids, and lactones including matricin and apigenin	Strongest beneficial effects were observed for the aggression and risk aversion subscales after three weeks of treatment in the probiotics group. Selective for cognitive reactivity to depression and not for self-reported symptoms of depression and no side effects were reported.
Ahmed et al. [21]	2013	Efficacy in altering microflora in Crohn's colitis	Trevis capsule (Chr. Hansen, Hørsholm, Denmark) that contains four strains of probiotics, *Lactobacillus acidophilus LA5, Lactobacillus delbrueckii *subsp.* bulgaricus LBY-27, Bifidobacterium animalis *subsp.* lactis BB-12, *and *Streptococcus thermophilus STY-31*) + prebiotic (oligofructose)	Placebo	Statistics showed no significant modulation of microbiome between cases and controls, the results are not only insignificant but also erratic, and the median age of patients is 62 years. No side effects are discussed; however, the population size is small and old aged, and there is no washout period between switching the groups.
Fedorak et al. [22]	2014	Postoperative recurrence. Multistrain probiotics are used since single strains are ineffective with the existing evidence. Patients aged 16 or above who underwent ileocolonic surgical resection with a small-intestine-to-colon anastomosis	VSL#3 which is a mixture of eight bacteria that contains four strains of *Lactobacillus *(*L. paracasei DSM24733, L. plantarum DSM24730, L. acidophilus DSM24735, and L. delbrueckii *subsp.* bulgaricus DSM24734*), three strains of *Bifidobacterium *(*B. longum DSM24736, B. breve DSM24732,* and *B. infantis DSM24737*),and one strain of *Streptococcus salivarius* subsp.* thermophilus DSM24731*	Placebo, codeine, loperamide, diphenoxylate, and cholestyramine for diarrhea. Probiotics given for 90 days overall	At day 90 (after phase 2), the proportion of patients with endoscopic recurrence is 9.3% in VSL#3-treated group and 15.3% in the placebo group with p=0.19, and the rate of recurrence is also not significant (p=0.82). At day 365 (after phase 3), there was no severe recurrence in 89.6% of previous VSL#3 users continuing VSL#3 in phase 3, and there is no severe recurrence in 71.4% patients from the placebo group who are taking VSL#3 in phase 3 but did not take VSL#3 in phase 2. Severe recurrence rate in early VSL#3 group is 20.5% and in early placebo group is 42.1%. Colonic mucosal cytokine release is also less in patients who started VSL#3 immediately and high in immediate placebo groups (took VSL#3 lately). Side effects: In the early placebo + late VSL#3 group, two patients had small bowel obstruction due to adhesion, one patient faced worsening of disease, one patient experienced postoperative wound infection, there was one case of ventral hernia repair, and there was one case of traumatic stabbing, whereas only one case of post-op wound infection is seen in the early + late VSL#3 group.
Bourreille et al. [23]	2013	Disease relapse in patients with CD who underwent remission during therapy with steroids or aminosalicylate. 52-week randomized, double-blind, placebo-controlled trial aimed at evaluating the prophylactic efficacy of *Saccharomyces boulardii* in CD patients. It is a one-year study.	*Saccharomyces boulardii, a *nonpathogenic probiotic yeast	Placebo	By week 52, 80 patients had experienced relapse, 38 (47.5%) in the *S. boulardii* group and 42 (53.2%) in the placebo group (*P*=0.5), and there was no difference in relapses occurring in 32 patients (54.2%) receiving *S. boulardii* and 38 patients (57.6%) receiving placebo (*P*=0.7). The median (range) time to relapse was not statistically different between patients treated with *S. boulardii* or placebo. The percentage of relapses during the weaning period of the initial flare-up treatment was similar in the two groups, 18 (22.5%) in the *S. boulardii* group and 24 (30.4%) in the placebo group (*P*=0.26). A total of 14 patients (8.8%) relapsed after treatment discontinuation, five in the *S. boulardii *group and nine in the placebo group. The change in CDAI is also not significant (P=0.99); the interaction between treatment group and smoking status was statistically significant (P=0.016). Nonsmokers given placebo had more relapses (72.0%) than those treated with *S. boulardii* (34.5%). Probiotics made no difference between nonsmokers and former smokers; however, when adjusting for stratification factor, nonsmokers treated with *S. boulardii* were less likely to relapse than those receiving placebo (p=0.006). Three variables were associated with a relapse which are use of corticosteroids as initial flare-up therapy, extraintestinal manifestations, and location of the disease. No significant changes in ESR were noted. 215 AEs were reported in 94 patients (diarrhea, arthralgia, constipation, and abdominal pain were commonly seen), but no significant discrepancy is seen between probiotics and placebo groups. Overall, 21 serious AEs occurred with no difference between the two groups.

**Table 4:** Animal and in vitro studies DSS: dextran sulfate sodium; IBD: inflammatory bowel disease; DAIS: Disease Activity Index Score; NO: nitric oxide; CD: Crohn's disease; MPO: monoperoxide oxidase; LPS: *Lactobacillus plantarum* strains; PGN: peptidoglycan; iNOS: inducible nitric oxide synthase; IL: interleukin; ICR mice: Institute of Cancer Research mice; BW: body weight; CCR6-CCL20 axis: chemokine receptor 6-chemokine ligand 20 axis; B1: *Bifidobacterium animalis* spp. *Lactis Bi1*; B2: *Bifidobacterium breve Bbr8*; L1: *Lactobacillus acidophilus LA1*; L2: *Lactobacillus paracasei 101/37*; 6MP: 6-mercaptopurine; TNF: tumor necrosis factor; MDM: monocyte-derived macrophage; HD: healthy donor; MODC: monocyte-derived dendritic cells; HBI: Harvey-Bradshaw Index; *AIEC LF82: adherent invasive E. coli LF82*; COX-2: cyclooxygenase-2; PGN: peptidoglycans; IECs: intestinal epithelial cells; CCR6-CCL20 axis: chemokine receptor 6-chemokine ligand 20 axis; HD: healthy donors; UC: ulcerative colitis

Author of study	Year of publication	Subjects	Sub-topic	Probiotics studied	Placebo/other drugs	Result and side effects
Kim et al. [24]	2020	18 mice. Mice were randomly divided into three groups as follows: a control supplied with normal drinking water, a DSS-treated group followed by oral administration of vehicle, and a DSS-treated group treated with *L. plantarum CBT LP3* daily for seven days following DSS administration	Inflammation, symptoms, and histopathology	L. plantarum CBT LP3	Drinking water containing DSS which induces colitis that resembles IBD	Very significant p-values were seen in reducing DAIS, substantial reduction was seen in inflammatory infiltrates, normal intestinal epithelial histology is restored, BW loss is controlled and colon length is increased, but the survival rates are statistically insignificant. DSS treatment increased pro-inflammatory cytokines especially IL-1J and IL-1B and NO production, whereas* L. plantarum CBT LP3* substantially reduced them. Drastic increase in the proliferation and count of Th2 lymphocytes and T-regulatory cells was observed in mice given probiotic. There was almost no change in both types of macrophage count. P-values are significant but are not written clearly in the paper, and no side effects were mentioned in the study.
Sivignon et al. [25]	2015	T84 cells and enterocytes from CD patients	Disease severity index, histopathology, immunomodulation, and inflammation	*Saccharomyces cerevisiae CNCM I-3856 *(*yeast*)	*AIEC LF82*. DSS to induce colitis in subjects	Effects of treating enterocytes/mice incubated or already treated with *AIEC LF82 Saccharomyces cerevisiae CNCM I-3856* (*yeast*) are as follows: ​Pre-incubation protocol, wherein *E. coli* were added one hour after incubating colitis cells along with *yeast* in vitro, showed the results as follows: With a *yeast* count of 5x10^6 ^yeasts/ml, the *E. coli* adhesion to intestinal cells came down to 25.9+/- 2.8% from 100%, and with a *yeast* count of 10^7 ^yeasts/ml, it came down to 11.8+/-3.9%. On the other hand, residual invasion level reduction is with 5x10^5^ yeasts/ml to 42.7+/-10.6% and with 10^7^ yeasts/ml to 6.7+/-2.7%. In co-incubation protocol with 10^7 ^yeasts/ml, the adherence decreased to 16.4+/-6.7%. In post-incubation protocol, residual adherence with 10^7^ yeasts/ml is reduced to 60.1+/-8.5%. Co-incubation with eight subspecies of *E. coli *(*LF9, LF15, LF16, LF31, LF50, LF54, LF65, and LF110*) showed that the fall in adherence levels was between 17.5% and 48%. *AIEC* adherence to brush border cells of primary ileal enterocytes in three patients without treatment got significantly reduced after treatment with *yeast*. This change is also dose dependent; 2.5x10^7 ^yeasts/ml showed great results. Other effects are gut colonization and disease activity decreased by day 5. By day 7, these changes became more significant. DAIS: significant improvement in weight loss prevention (P<0.05) and colitis. By day 7, it came to normal limits like that of control mice. Four of six mice with LF82 infection showed blood in stools; none in *yeast*-treated group. At day 7, the survival rate of LF82-infected mice is 30%, and *yeast*-treated mice is up to 70%. Histological scores: Untreated is 5.6+/-0.9, treated is 2.4+/-0.5, and p-value is <0.01 (nearly same as normal mice). Massive neutrophilic infiltration with transmural involvement, lymphoepithelial lesions, and mucosal erosions are seen in the non-treated group, but not seen in the treated group. MPO activity is altered but not significant. Intestinal permeability restored to almost basal levels. The overexpression of CLAUDIN-2 (a pore-forming tight junction protein) associated with plasma membrane is resolved. Effects of treating infected mice with cell wall derivatives of yeasts rather than live yeasts: Less severe BW loss seen (direct yeast is 90.2% and with derivatives is 83.9%) and DAIS is not significant after days 3 and 4.
Choi et al. [26]	2019			*Lactobacillus plantarum *(*CAU1045, CAU1054, CAU1055, CAU1064,*and* CAU1106*), *Weissella viridescens CAU1224, Lactobacillus sakei *ssp.* sakei CAU1273, Lactobacillus salivarius CAU1301*	Controls	Suppression of NO synthesis compared with the LPS-stimulated NO controls: *CAU1064* (46% inhibition),* CAU1031* (44.8%), *CAU1273* (41.4%), *CAU1054* (38.2%), *CAU1106* (32.3%), *CAU1055* (31.0%), *CAU1045* (23.5%),and *CAU1224* has no effect. P-values of suppression of various components: (i) NO synthesis, <0.001; (ii) iNOS and COX-2 expression, <0.001; and (iii) cytokines IL-6 and TNF-α inLPS-induced RAW264.7 cells, <0.001. Efficacy of PGN of probiotics in the suppression of iNOS expression: *CAU1055* (45.8% inhibition),* CAU1064* (45.6%), *CAU1054* (45.1%),* CAU1045* (36.6%), *CAU1301* (35.4%), *CAU1106* (35%), and *CAU1273* (16.8%). Inhibition of COX-2 expression in LPS-induced RAW264.7 cells: *CAU1064* (40% inhibition),* CAU1106* (39.5%), *CAU1055* (33.7%),* CAU1054* (33.3%), *CAU1045 *(20.6%), *CAU1301* (20.3%), and *CAU1273* (19.6%). Effects on TNF-α suppression: *CAU1055* (83% inhibition), *CAU1064* (83%), *CAU1054* (77.7%), *CAU1106 *(74.6%),* CAU1301* (67.2%), *CAU1224* (55.7%), *CAU1273* (47.2%), and *CAU1045* (37.8%). Effects on IL-6 suppression: *CAU1106 *(92.8% inhibition), *CAU1055* (91%), *CAU1064* (90%),* CAU1054* (89%), *CAU1045* (80.5%), *CAU1301* (76.6%), and *CAU1273* (49.8%). In ICR mice *L. plantarum CAU1055* significantly increased BW (32.78±0.41 g) compared with the DSS-treated group (30.53±0.87 g), while no other strains showed this effect. Effect on DSS-induced colitis in ICR mice: Only *L. plantarum CAU1055* significantly increased BW (32.78±0.41 g) compared to the DSS-treated group (30.53±0.87 g), and reduced colon length was most strongly (from 6.7±0.19 cm to 9.27±0.08 cm). Effect of* L. plantarum CAU1055* on inflammatory mediators in DSS-induced colitis model: mucosal damage and inflammatory cell infiltration are significantly reduced.
Leccese et al. [27]	2020	Blood samples of nearly 18 patients. Patients who were not receiving immunosuppressive therapies and/or antibiotics were selected according to the disease activity, using HBI for CD	Immunology (counteracting the virulence of *AIEC*)	B1, B2, L1, L2	*AIEC LF82-AIEC LF82. Staphylococcus epidermidis ATCC-155 *(a non-probiotic gram-positive bacterium). 6MP is an anti-inflammatory drug and a strong inhibitor of *AIEC LF82*	In the experiments, the effects of *AIEC LF82* invasion of IECs and CCR6-CCL20 axis were tested. Again, to verify that LF82 inhibition is not simply due to any bacterial strain, co-infection experiments with* S. epidermidis ATCC155* and also with 6MP were conducted, and the results showed that L1, L2, B1, and B2 significantly reduced *AIEC LF82* adhesion to human colorectal adenocarcinoma cell line with epithelial morphology (HT29 cells) by blocking specific adhesion determinants and enhanced biofilm formation. Their the p-values are as follows: decrease in cell-associated *AIEC LF82* after three hours, <0.05; decrease in percentage intracellular *AIEC LF82*, <0.01; decrease in IL-8 and CCL20 in pg/ml, <0.001; and modulation in the invasion and survival of *AIEC LF82* inside IECs after three and seven hours of infection showed that 6MP has a great effect (p<0.001).* L1* and* L2* are effective but less than 6MP, *B1* and *B2* are still less effective, and *S. epidermidis* has no effect. Reduction in the induction of IL-8 in pg/ml are as follows: L1, from 475.3 to 139.7; L2, from 475.3 to 123.7; B1, from 475.3 to 102.1; B2, from 475.3 to 86.77; and *S. epidermidis*, from 475.3 to 383.1. No suppressive effect on CCL20 production was seen. Effect on the phagocytosis of *AIEC LF82*: *L1, L2, B1, B2*, and 6MP have no effect on *AIEC LF82* phagocytosis by CD-derived MDM. Probiotics could not effect intracellular concentrations of *AIEC LF82* unlike in ulcerative colitis.* AIEC LF82 *survival within CD-derived MDM after treating with *S. epidermidis* is high, with 6MP decreased to incredibly low levels. Probiotics treatment reduced TNF-α secretion in AIEC-infected MDM from HD but not from CD which was confirmed by TNF-α/IL-10 ratio, higher in HD than in CD and UC. Exception to this ineffectiveness of probiotics is B2 (P<0.0001). In CD-derived MDM, TNF-α levels remained the same or increased especially with B2 from 417.2-900.7, and B1 and B2 inhibited IL-10 secretion significantly (P<0.0001). *S. epidermidis* directly increased TNF-α and IL-10 release. 6MP decreased cytokine release in HD (P<0.01) and decreased IL-10 release in CD; TNF-α levels not changed (p=0.9380). Probiotics reduced *AIEC LF82* survival and production of pathogenic helper T-lymphocyte-7 (Th17) polarizing in MODC in HD and UC, but not in CD. Polarizing cytokines (IL-12p, 23, 1 beta) stimulate Th1 and Th17 differentiation and expansion, which is an immunological characteristic of CD. Only* L2* and *B2* showed effect on *AIEC LF82* uptake by MDM and significantly reduced the number of phagocytosed LF82 in MODC in CD patients (P=0.0068 and 0.001). 6MP strongly inhibited *AIEC LF82* uptake in MODC of HD patients or IBD patients. *S. epidermidis* has no effect. In contrast to HD and UC, in CD, L2 and B2 failed to maintain lower intracellular LF82 bacterial load as measured from eight to 24 hours post infection from -68.9% to 37.7% with* L2* and from 70% to 38.7% with *B2*. No change with *S. epidermidis. *No change with 6MP. Pattern of cytokine secretion by *LF82*-infected MODC depends on the origin of MODC. In CD-derived MODC, the ratio between the amount of IL-1 beta, IL-23, and IL-10 and the number of intracellular LF82 cells after 24 hours of infection is very high compared to HD, while IL-12 level is very low. *B2* in CD-derived MODC sharply decreased IL-23 secretion (P=0.0099), reduced IL-1 beta secretion significantly (p=0.03), and had no effect on IL-10. 6MP shows non-specific impairment of pro-inflammatory and anti-inflammatory substance release. 6MP inhibits IL-12 only in CD-derived MODC.

**Table 5:** Review of various reviews and meta-analysis RCT: randomized controlled trial; CD: Crohn's disease; CDAI: Crohn's Disease Activity Index; 6MP: 6-mercaptopurine; SCFA: short-chain fatty acids; PR: postoperative recurrence; cfu: colony-forming units

Author of study	Year of publication	No of patients/subjects involved	Focused sub-topic	Probiotics/intervention studied	Placebo and/or other drugs given	Result and side effects
Chen et al. [28]	2021	289 patients involved in RCTs	Remission and recurrence	VSL#3 which is a mixture of eight bacteria that contains four strains of *Lactobacillus* (*L. paracasei DSM24733, L. plantarum DSM24730, L. acidophilus DSM24735,* and *L. delbrueckii* subsp. *bulgaricus DSM24734*), three strains of *Bifidobacterium *(*B. longum DSM24736, B. breve DSM24732,* and *B. infantis DSM24737*), and one strain of *Streptococcus salivarius* subsp.* thermophilus DSM24731. S. boulardii. L. johnsonii LA1. L. rhamnosus GG*	Placebo	CD recurrence reviewed from four studies that include 289 subjects (145 probiotic users and 144 other drug users) showed statistically insignificant results with a p-value of 0.52. In remission, the probiotics showed some effects, and no side effects were mentioned.
Dore et al. [29]	2019	178	Side effects	*Lactobacillus rhamnosus GG, L. johnsonii LA1, B. longum*+Synergy (Hydrofarm, Pennsylvania, United States)	Placebo	Study 1: No side effects are mentioned where 12 billion cfu/day probiotics was given for 52 weeks. Study 2: 2x10^9^ cfu/day for six months was given. Abdominal pain and unpleasant taste were reported. Study 3: 2x10^11 ^cfu/twice daily with 6 grams of fructooligosaccharides or inulin was given for six months. Out of 35 patients, only two patients could not tolerate synbiotics.
Lorentz and Müller [30]	2022	32 subjects after removing duplicates. 482 in total, out of which 450 are covered in other studies	Remission and recurrence	*S. boulardii, L. rhamnosus GG, S. boulardii, L. johnsonii LA1, *Symprove (a mixture of *L. rhamnosus*,* L. plantarum*,* L. acidophilus,*and *E. faecium* in barley extract, a water-based preparation)	Placebo and mesalazine	Guslandi et al. studied the efficacy of *S. boulardii* with mesalazine and only mesalazine for six months. Low recurrence is seen in the probiotics plus mesalazine group in adulthood. The disease recurrence in probiotics+mesalazine users is 6.25%, whereas in mesalazine-only users, it is 37.5%. All other studies showed no benefits with the probiotics.
Limketkai et al. [31]	2020	45	Remission in symptomatic CD	*L. rhamnosus GG, Bifidobacterium* *longum,* and 6 grams Synergy	Placebo. Cornstarch placebo.	The first study included 11 participants who had CDAI between 150 and 300, who were treated with one week of corticosteroids and antibiotics prior to the study, and who were then treated with *L. rhamnosus GG* (two billion cfu per day) or cornstarch placebo. Other study with 35 participants with CDAI of 150-450 received *Bifidobacterium longum* and 6 g Synergy or placebo, and the results showed no significant decline in disease activity index (I^2^=0%). No side effects were reported except intolerance in two patients, but only a small population size had been studied.
Shen et al. [32]	2014	Total 349 out of which 306 are already studied in this paper. 43 exclusive subjects	Remission	*E. coli* Nissle	Placebo. Placebo+aminosalicylates, 6MP, azathioprine, corticosteroids	Three subgroup analyses (Malchow, Schultz, and Steed) suggested no significant benefit in CD (*P*=0.35, RR=0.89), seven studies suggested no significant change in clinical relapse (*P*=0.71, RR=1.09), and three trials (Prantera, Marteau, and van Gossum) reported insignificant endoscopic relapse rates (*P*=0.75, RR=1.08). In maintaining therapy for CD, the results from six trials (Malchow, Prantera, Schultz, Marteau, van Gossum, and Steed) suggested that administration of probiotics and placebo had no significant difference (*P*=0.67, RR=0.89), with little heterogeneity indicated (*P*=0.73, I^2^=0%). Although *Bifidobacterium*, *E. coli*,and *Lactobacillus* were used for maintaining therapy in different trials, they did not show any favorable effect over controls in patients with CD.
Zhuang et al. [33]	2021	CD 709, Controls 98, Total 807	PR	*Actinomyces*, *Bacteroides*, *Bifidobacterium,* *Blautia*, *Clostridium*, *Coprococcus*, *Dorea*, *Enterococcus,* *Eubacterium*, *Faecalibacterium*, *Fusobacterium*, *Lachnobacterium*, *Lachnospira*, *Lactobacillus*, *Odoribacter*, *Paraprevotella*, *Phascolarctobacterium*, *Prevotella*, *Ruminococcus*, *Streptococcus*, *Veillonella*	Antibiotics and corticosteroids	Mucosa-associated microbiota in CD patients exhibited reduced bacterial richness and diversity, while there was a clustering of patients and healthy controls. Machiels established that fecal and mucosal microbiota constitute different ecological environments before surgery. No bacterial taxon altered counts consistently. The alterations in mucosal microbiota are more significant in *Bacteroidetes* and *Firmicutes* phyla, but less significant in *Proteobacteria* phylum; also, the expansion of *Fusobacteria*, a putative aggressive phylum, was also observed. The factors influencing bacterial populations following ileocolonic resection are substantial catabolic stress, retrograde flow of colonic contents, inflammatory changes involved in intestinal wound healing, and altered immune function. At the time of postoperative endoscopy, the population of *Actinobacteria*, a proteolytic bacterial phylum with the capacity to invade and exacerbate inflammation, was depleted in patients with CD; however, the phylum *Fusobacteria* maintained its higher relative abundance, while the alterations in the phylum *Bacteroidetes* count were inconsistent. Higher abundance of *Enterococcaceae* and *Fusobacterium* and lower abundance of *Lachnospiraceae* and *Faecalibacterium* existed in both postoperative mucosal and fecal microbiota in CD patients. As fecal- and mucosal-associated microbiota constitute different ecological environments, they have different alteration trends. Notably, the presence of mucosal bacterial genera, such as *Bacteroides*,* Prevotella*, and *Parabacteroides*, which are associated with saccharolytic metabolism, has been correlated with increased remission compared to the presence of bacterial genera, such as *Enterococcus* and *Veillonella*, which are associated with fermentation and lactic acid production. However, no specific bacterial taxa are consistently different between CD patients with or without PR in any of the included studies. Several commensal bacteria with lower relative abundance are known to exert an anti-inflammatory effect by decreasing colonic pro-inflammatory cytokine synthesis and inducing anti-inflammatory cytokine secretion. In addition, patients with recurrent CD retain microbiota that favors proteolytic-fueled fermentation and lactic acid production, while CD patients in remission retain a predominantly saccharolytic and SCFA-producing microbiota. Furthermore, changes in the ecology of depleted SCFA-producing bacteria may permit the expansion of pathogenic bacteria through luminal environmental perturbation (deviation from normal). Till date, no study has evaluated the role of the metabolomic profiling of gut microbiota at the time of surgery or at the postoperative follow-up in the identification of metabolites that may be associated with CD recurrence after intestinal resection. The number of published studies investigating the involvement of gut microbiota both in monitoring the postoperative disease progression and in assessing the response of CD patients to a treatment is surprisingly low. There are only four studies that provide information on the potential of gut microbiota to predict the PR of CD, but their results were too heterogeneous to make a confident conclusion regarding a microbial biomarker. High abundance of bacteria from the *Proteobacteria* phylum (e.g., *Proteus* and *Ralstonia*) as well as in *Ruminiclostridium gnavus* (*Gammaproteobacteria*) and *Corynebacterium* and the reduced abundance of several members of the *Firmicutes* phylum, particularly the *Lachnospiraceae* and the *Ruminococcaceae* families (e.g., *Faecalibacterium*,* Gemella*,* Phascolarctobacterium*,* Coprobacillus*,* unidentified Lachnospiraceae*, and *Dorea*), were predictive of endoscopic PR. Keshteli's study says distinctive urinary metabolomic profiling associated with *Bacteroidales *and* Gammaproteobacteria* has the potential to be used as a biomarker for the identification of CD patients who develop endoscopic disease recurrence after ileocolonic resection. Study by Campieri reported that the combination of a non-absorbable antibiotic (rifaximin) and a highly bacterial concentrated probiotic (VSL#3) is efficient in preventing the severe endoscopic recurrence of CD. Early postoperative use of antibiotics to prevent pathogenic recolonization followed by maintenance with the use of probiotics to establish a durable anti-inflammatory postoperative microflora may yield the greatest benefit with the least risk of disease recurrence in CD patients. Unfortunately, the efficacy of fecal microbiota transplants in preventing the PR of CD remains unknown. Exclusive probiotic therapy is not found to be effective in preventing PR. Antibiotics are superior over probiotics in this regard. Insufficient trials.

Discussion

Almost 2023 cases are studied from all the 16 papers, and only very few probiotic preparations are found worth of using in adult CD patients based on the available latest evidences. There are certain probiotics that showed mind-relaxing results. 

Yılmaz et al. [19] with 45 subjects in the year 2019 established that consumption of kefir, a fermented milk product, in adults showed excellent symptom relief which is statistically significant. It also improved quality of life and reduced inflammation. Moreover, its consumption had no lactose intolerance-related effects; hence, kefir is safe, affordable, and very effective with short-term consumption which proves it to be an asset for CD patients. We explosively recommend further large population-sized clinical trials with kefir as we anticipate the same results. The effect of kefir in long-term consumption has to be studied.

On the other hand, Bourreille et al. [23] in the year 2013 studied 125 cases and proved that *Saccharomyces boulardii* had no side effects. Location of the disease in the gastrointestinal tract of the patients has no influence on the risk of relapse. However, this study excluded severe CD patients, and mucosal healing is not studied systematically. Biological parameters were used to assess inflammation. *S. boulardii* at a daily dose of 1 gram, although safe and well tolerated, did not show any beneficial effect as a preventive remedy in patients with moderate and severe CD. No effect on dwindling side effects was noted.

Bjarnason et al. [18] in the year 2019 with 250 participants showed that Symprove (an admixture of *Lactobacillus rhamnosus NCIMB 30174*,* Lactobacillus plantarum NCIMB 30173*,* Lactobacillus acidophilus NCIMB 30175*, and *Enterococcus faecium NCIMB 30176*) taken for four weeks in asymptomatic CD patients is not effective in symptom relief and improving quality of life and perfecting emotional symptoms, but minimally effective in preventing re-occurrence. Wu et al. [20] in the year 2021 with 46 subjects reported that *Lactobacillus thermophilus*,* Bifidobacterium longum*,* Enterococcus faecalis*,and* Bacillus licheniformis* are safe and have strongly controlled the diarrhea symptoms through regulating the gut microbiota in the CD patients between 22 and 54 years of age and also had better cognitive reactivity to sad mood in three weeks.

Ahmed et al. [21] in the year 2013 with very small population size demonstrated that Trevis capsule that contains four strains of probiotics, *Lactobacillus acidophilus LA-5*,* Lactobacillus delbrueckii *subsp.* bulgaricus LBY-27*, and *Bifidobacterium* subspecies is safe, but had no beneficial effects on old-aged people in CD in altering colonic microflora. Fedorak et al. [22] in the year 2014 with 119 subjects showed that commencing VSL#3 soon after surgery might not show better results after few months, but prevents recurrence with consumption for one year and crucial in easing numerous side effects in 16-year-old or above aged patients who underwent ileocolonic resection with a small-intestine-to-colon anastomosis. VSL#3 is safe in CD. Kim et al. [24] in the year 2020 demonstrated that, in colitis-induced mice, *L. plantarum CBT LP3* is largely effective in alleviating disease activity, weight loss, and inflammation. Histopathological damage is very much reduced. It is effective in preventing relapse as well. Although the population size is small, based on the results of this study, it is recommended to conduct analogous experiments in animals with bigger population size, and then with at most safety precautions, human trials could be conducted to demonstrate the use of *L. plantarum CBT LP3* in CD colitis. Side effects are to be studied in human trials.

In 2015, Sivignon et al. [25] studied the efficacy of *Saccharomyces cerevisiae CNCM I-3856* (*yeast*) in preventing colitis induced by *adherent invasive Escherichia coli (AIEC) LF82* in mice, and very significant results were obtained; pre-incubation results are more prominent than co-incubation and post-incubation effects. Such appreciable results are not seen with the cell wall components of *AIEC LF82*. Significant results are observed in reducing disease activity index and quality of life. Human trials are needed to study the side effects and further confirmation of findings: if *S. cerevisiae* yeasts could prevent CD in patients abnormally expressing CEACAM6 at the ileal mucosa. Study by Choi et al. [26] in 2019 concluded that in lipopolysaccharide-induced RAW264.7 cells and a dextran sulfate sodium (DSS)-induced colitis animal model, the effects of probiotics are as follows: *Lactobacillus plantarum CAU1064*, *CAU1031*, *CAU1273*, and *CAU1054* strains have nitric oxide (NO) and cyclooxygenase-2 (COX-2) suppression potential. Peptidoglycans of *Lactobacillus plantarum CAU1055*, *CAU1064*, *CAU1054*, *CAU1045*, *CAU1301*, and *CAU1106* have inducible nitric oxide synthase (iNOS) suppression potential. *Lactobacillus plantarum CAU1064*, *CAU1106*, *CAU1055*, and *CAU1054 *can inhibit COX-2 inhibition potential. *Lactobacillus plantarum CAU1055*, *CAU1064*, *CAU1054*, *CAU1106*, *CAU1301*, *CAU1224*, *CAU1273*, and *CAU1045* have very significant TNF-α suppression effect. *Lactobacillus plantarum CAU1106*, *CAU1055*, *CAU1064*, *CAU1054*, *CAU1045*, *CAU1301*, and *CAU1273* have excellent IL-6 suppression potential. *Lactobacillus plantarum CAU1055 *significantly increased body weight in ICR mice, and it also had greatly ameliorated colon length reduction. *Lactobacillus plantarum CAU1055* also significantly reduced mucosal damage and inflammatory cell infiltration in in DSS-induced colitis. These results are impressive; therefore, strains 1055, 1064, 1106, and 1054 need to be tested on CD and related colitis in human beings since these four strains are proven to be significantly effective than remaining strains.

In vitro study by Leccese et al. [27] in the year 2020 suggests that the efficacy and safety of* Bifidobacterium animalis *spp. *Lactis Bi1* (B1),* Bifidobacterium breve Bbr8 *(B2), *Lactobacillus acidophilus LA1* (L1),and* Lactobacillus paracasei 101/37* (*L2*) (especially B2) should be studied in *AIEC LF82*-induced CD by conducting RCTs as they significantly controlled the adhesion of *AIEC LF82* and enhance biofilm formation. Chen et al. [28] in the year 2021 reviewed papers with 289 CD patients and showed that VSL#3 that contains *Lactobacillus* strains (*L. paracasei DSM24733*,* L. plantarum DSM24730*,* L. acidophilus DSM24735*, and *L. delbrueckii *subsp.* bulgaricus DSM24734*), three strains of *Bifidobacterium* (*B. longum DSM24736*,* B. breve DSM24732*, and *B. infantis DSM24737*), and one strain of *Streptococcus salivarius *subsp.* thermophilus DSM24731 *showed no significant results. The results showed that *S. boulardii*,* L. johnsonii LA1*,and* L. rhamnosus GG* have no remarkable goods in the remission and recurrence of CD. Study by Dore et al. [29] in the year 2019 conducted a systematic review that involved 178 patients to estimate the side effects of *Lactobacillus rhamnosus GG*, *L. johnsonii LA1*,* B. longum*, and *Synergy* and showed that, if the unpleasant taste is changed using required techniques, oral probiotics are more comfortable and much safe in CD. Lorentz and Müller [30] in the year 2022 conducted a systematic review with 482 subjects involved, out of which 32 were CD cases. This review established that *S. boulardii* reduces recurrence in mesalazine users in CD. Limketkai et al. [31] in the year 2020 conducted a systematic review that involved 45 subjects and showed that *Bifidobacterium longum* and *Synergy* are ineffective in CD remission.

A meta-analysis conducted by Shen et al. [32] in the year 2023 which involved 43 subjects, studied effects of probiotics on the remission of CD, and showed that *Bifidobacteria*, *E. coli*, and *Lactobacillus* were used for maintaining therapy in different trials did not show any favorable effect over controls in patients with CD. No significant effect on problem remission was seen. Zhuang et al. [33] in the year 2020 conducted a systematic review with 809 patients to check the role of several probiotics in postoperative recurrence, but no significant results were retrieved. Exclusive probiotic therapy is not found to be effective in preventing postoperative recurrence. Antibiotics are found to be superior to probiotics in this regard. Trials are regarded to be inadequate.

## Conclusions

This systematic review studies the safety and efficacy of various probiotics in CD remission and postoperative recurrence and their side effects from the latest available research from several databases. We excluded CD patients that developed cancer, pregnant women, and ICU patients. The latest available quality data from the last 10 years shows that kefir which is a fermented dairy product is an asset for adult CD patients. *L. thermophilus*,* B. longum*,* E. faecalis*, and *B. licheniformis* are safe and have strongly controlled the diarrhea in patients between 22 and 54 years age and also have better cognitive reactivity to sad mood in three weeks. *VSL#3* prevents recurrence and side effects in adults undergoing ileocecal resection. These results in turn must be tested in large population-sized trials in order to establish their effects on a large scale. Few probiotics proven very effective in animal and in vitro studies are to be tested in human trials, and there is a big scope based on these results that testing them in human trials is worth sought after. *S. boulardii* reduces recurrence in mesalazine users in CD. The unpleasant taste of oral probiotics could be changed to increase their use. Hence, we reckon the presentation of evidences and interpretation of effects are precise enough albeit there are less number of clinical trials and very less observational studies available regarding the role of probiotics in CD in human beings.
